# Evolution of altruistic punishments among heterogeneous conditional cooperators

**DOI:** 10.1038/s41598-021-89563-z

**Published:** 2021-05-18

**Authors:** Balaraju Battu

**Affiliations:** Department of Political and Social Sciences, European University Institute, San Domenico di Fiesole, 50014 Florence, Italy

**Keywords:** Ecology, Evolution, Environmental social sciences

## Abstract

It has been known that altruistic punishments solve the free rider problem in public goods games. Considering spatial structure and considering pure strategies significant advances have been made in understanding the evolution of altruistic punishments. However, these models have not considered key behavior regularities observed in experimental and field settings, where the individuals behave like conditional cooperators who are more willing to donate and are also more willing to punish free riders. Considering these behavioral regularities, without imposing a spatial structure on the population, I propose an evolutionary agent-based model in which agents behave like conditional cooperators, each agent’s donation conditional on the difference between the number of donations in the past and the threshold value and the propensity value of the agent. Altruistic punishment depends on the difference between the threshold value of the focal agent and the randomly matched another agent. The simulations show that, for certain inflicted costs of punishments, generous altruistic punishments evolve and stabilize cooperation. The results show that, unlike previous models, it is not necessary to punish all free riders equally; it is necessary to do so in the case of the selfish free riders but not in the case of negative reciprocators.

## Introduction

Unlike other species, humans cooperate with genetically unrelated individuals, even if these are individuals they have never met in the past. In the literature several mechanisms have been proposed to understand the suitable conditions with which to establish cooperation in dyadic interactions^1–7^. The mechanism focuses on the conditions required to offset the current cost of cooperation with future obtainable benefits. Unlike dyadic interactions, in public goods provision, an individual incurs personal costs by contributing to the public good and the benefits are shared among the group members because public goods are ‘non-excludable,’ i.e., each individual can share them equally, irrespective of their contributions^8^. Contrary to the assumptions of standard economic theory, laboratory^9–11^ and field studies^12^ suggest that, in repeated public goods games, the majority of the population is willing to punish the free riders, even at incurring substantial personal costs^13^. Remarkably, the punishments are implemented even if the interactions are anonymous, with no gain in personal payoff and reputation^11^. Neurobiological studies show that altruistic punishment has been linked to the reward centers of the brain, suggesting that in the past such behavior was rewarded and evolved in populations^14^. An altruistic punisher incurs personal costs by punishing free riders and the free riders gain a relatively higher payoff than the punishing individuals and cooperators. In evolutionary biology costs and benefits are measured in terms of fitness, therefore the altruistic punisher reduces his/her fitness in order to improve the group’s fitness. Altruistic punishments are a powerful mechanism with which to establish cooperation in social dilemmas. Why altruistic punishments proliferate in human societies has been an evolutionary puzzle because natural selection supposes favoring free riding rather than costly punishments.

In the last decade, significant advances have been made to understand the evolution of punishments in social dilemmas. It has been proposed that pool^15–18^ and peer^19–22^ punishments provide a solution to free rider problem in public goods games. In general, a population consists of cooperators, free riders, and punishers, it is hard to evolve punishment strategies when the individuals interact in a well-mixed manner, but spatial structure allows for the evolution of diverse punishment strategies and can establish stable cooperation^21,23–27^. For instance, conditional strategies such as conditional punishments^28^, class dependent strategies^24^, sharing responsibility for punishments^25^, and social diversity^23^ also protect a population from free riding in public goods games. It has been shown that when the population consists of altruists, unconditional defectors or free riders, non-participants, and altruistic punishers, the altruistic punishers dominate the population; but when the individuals in the population are averse to losing their reputation due to free riding^29,30^ and punishers separate from the cooperators and punish the free riders^20,21^, the population can solve the second order free rider problem. The former method requires an additional layer of indirect reciprocity imposed on a public goods game and the latter method requires spatial structure to separate punishers from cooperators and punish free riders.

Without considering the spatial structure of the population, the evolution of altruistic punishments in public goods games has been studied and explained in two important classes of model based on group selection^31–33^ and individual selection^34^. The group selection models have shown that in certain conditions altruistic punishment does evolve and is evolutionarily stable when the punishment is common, but this does not explain how the altruistic punishment emerges within the group or when individual selection operates within the group. In both classes of model, the cooperative strategies and punishment strategies do not depend on the past behavior of other individuals in their groups. The behavioral regularities observed in experimental public goods games^35,36^ and field studies^12,37^ suggest that individuals in the population behave like conditional cooperators and the population is heterogeneous in their conditional nature^35–38^. Further, there are no separate altruists and altruistic punishers and the individuals who are more willing to donate to the public good are also more willing to punish the free riders^9,37^ or are involved in costly monitoring of the group^12^. There are a few studies set in spatial public goods games, such as conditional punishments^28^ and class dependent strategies^24^, which consider the composition of the population and define punishment strategies, but in these studies the individuals are not conditional cooperators. Clearly, although these studies made significant contributions to understand the evolution of punishment in public goods games, these models did not consider some of the properties of conditional cooperators. In other words, in the current model, I address how altruistic punishment strategies evolve in the population of heterogeneous conditional cooperators who are more willing to donate and also more willing to punish free riders.

By considering the behavioral regularities observed in repeated public goods games in the field and experimental settings, I propose an evolutionary agent-based model with a population of heterogeneous conditional cooperators. Unlike the previous models^20,21,24,25,28,32–34,39,40^, in the proposed model, (*a*) the majority of the population behave like conditional cooperators, (*b*) the more individuals willing to donate are also more willing to punish free riders, and (*c*) the population is heterogeneous^12,36–38,41^. The proposed model involves three stages: individuals or agents make donations occasionally to a public goods game according to their conditional cooperative strategies. After a couple of rounds of the game, reciprocators potentially punish the potential free riders, and agents imitate successful role models' social behavior. In all three stages agents may commit mistakes. Whenever an agent donates, the agent incurs a certain cost and whenever an agent is punished the agent incurs inflected cost and inflected cost is more than the cost of the altruistic punishment. The simulations show that populations of heterogeneous conditional cooperators establish high levels of cooperation in repeated public goods games and the altruistic punishers dominate the population for certain inflected costs. The cooperation is stable against occasional mutations in the population. Further, for certain inflected costs, evolution favors generous altruistic punishment strategies more than strict punishment strategies, i.e., agents punish the occasional free riders with less frequency and the selfish free riders with higher frequency.

## Method

The above developed intuition is converted into an agent-based evolutionary model in the context of public goods provision. In the proposed evolutionary agent-based model^42^, all the agents play a linear public goods game by using conditional cooperative strategies, enforcing altruistic punishments based on relative differences in their cooperation tendencies, and imitating successful role models’ social behavior with certain errors. The process is iterated several thousands of generations.

### Population type

In the proposed model, the individuals or agents in the population behave like conditional cooperators and the population is heterogeneous in its conditional nature. The agents who are more willing to cooperate are also more willing punish to potential free riders. Each individual is born with an arbitrary conditional cooperative criterion (*CCC*) and a propensity, *β*. Both are positive values. The agents with higher *CCC* donate less frequently than the agents with a higher *CCC* for the given same amount of past cooperation levels. The same agents can cooperate or enforce altruistic punishments or free ride given the past cooperation levels in the population. *β* indicates the propensity to implement a conditional cooperative decision and imitate the successful role model’s social behavior. Each agent’s *CCC* value is drawn from a uniform distribution (0, *N*), where *N* is the population size, and *β* is drawn from a uniform distribution (0, 3). With *β* = 0 the actions of the individuals are random and with *β* = 3 the individuals behave like ideal conditional cooperators. With intermediate values the individuals behave like non-ideal conditional cooperators. The consideration is equal to the natural selection designing the conditional cooperative strategies. The combinations of *CCC* and *β* create heterogeneous populations with varieties of propensities. The consideration is close to the conditional nature of the population observed in experimental settings^12,36^.

### Conditional cooperative decision

The conditional cooperative decision of the agent is operationalized in the following way^43,44^. For instance, in the *r*th round, an agent *i* (with *CCC* = CCC_i_ value) donates to the public good with probability, *q*_*d*_,

1 $${q}_{d}= \frac{1}{1+\mathrm{exp}(-\left({n}_{C}-{CCC}_{i}\right){\upbeta}_{i})}$$

*n*_*C*_ indicates the number of donations in the (*r* − 1)th round. The parameter *β*_*i*_ controls the steepness of the probability function. For the higher *β*_*i*_, the agent is highly sensitive to the (*n*_*C*_*-CCC*_*i*_). For instance, as *β*_*i*_ → ∞, the q_d_ is sensitive to the sign of the (*n*_*C*_ -*CCC*_*i*_), i.e., if (*n*_*C*_ -*CCC*_*i*_) > 0 then *q*_*d*_ = 1 and if (*n*_*C*_* -CCC*_*i*_) < 0 then q_d_ = 0. Either with (*n*_*C*_* -CCC*_*i*_) = 0 or with β_i_ = 0 and both are zero, the agent donates to PGG with 50% time. In the model 0 < *β*_*i*_ < 3. With *β*_*i*_ > 2, the agent is more sensitive to the conditional rule. For β_i_ = 0, the agent ignores the rule and behaves randomly. In β_i_ < 2 and (*n*_*C*_* -CCC*_*i*_) < 2 the individual does not follow the conditional rule, occasionally. In this construction both agents with the same CCC value may act differently with different β_i_ values. For example, an agent with *n*_*C*_ = 25, *CCC*_*i*_ = 24, and *β*_*i*_ > 2 donates with probability close to one and *β*_*i*_ < 2, donates with the probability less than one. With a non-zero amount of cooperation in the zeroth round, very low *CCC* value agents potentially behave like altruists, middle *CCC* value agents behave like conditional cooperators and very high *CCC* value agents behave like free riders. In the model the population and the conditional cooperators are heterogeneous. The assumption is similar to the experimental and field observations in repeated public goods^9,12,36,37^.

### Public goods game

All the individuals in the population play a linear public goods game^37^ three times in each generation. In each round, each agent is given an initial endowment E and individuals potentially donate an amount u_i_ using Eq. (1). After all the individuals make their decisions, the collective amount is enhanced by a factor (α > 1) and the resulting public goods are distributed equally among all the agents, irrespective of their contributions towards public accounts. An agent’s total payoff from each linear public goods game is calculated using the following equation.

2 $${\uppi}_{Gi }=\left(E-{u}_{i }\right)+ \frac{\upalpha}{N} \left(\sum_{i=1}^{N}{u}_{i }\right), i=1,\mathrm{ 2,3},\ldots N, \; \upalpha >1, \frac{\upalpha}{N}<1$$

${\pi}_{Gi}$ is payoff of agent *i* from the game. The first term $\left(E-{u}_{i}\right)$ indicates the payoff from what was not contributed to the public goods (the private payoff). The second term indicates payoff from the public goods. Each unit donated becomes worth*α* > *1* unit. Due to the ‘non-excludable’ nature of public good, all the agents gain equal payoff from the public goods game. Clearly, all the individuals by donating can create an efficient PGG and by free riding increase his/her private payoff but reduce the group's payoffs. Overall, the free riders gain relatively higher payoffs than the donors if the game starts with few initial donations. Given the same amount of cooperation level, the higher CCC agents donate less frequently and gain relatively higher payoffs than the payoffs of the lower CCC agents who donate more frequently.

### Altruistic punishments

The assumptions of the model allow the following two rules: (i) the agents who are more willing to donate are also more willing to punish^9,12,37^. (ii) the agents punish the free riders more frequently than the occasional free riders^12,37^. The CCC value of an agent acts as a proxy measure of an individual's donation tendency and punishment tendency. The individuals enforce altruistic punishment based on the relative *CCC* difference of randomly matched pairs. An agent *i* (with *CCC*_*i*_) has the potential to punish another agent j (with *CCC*_*j*_) with probability ${q}_{p}$.

3 $${q}_{p}= \frac{1}{1+\mathrm{exp}(-\left({CCC}_{j}-{CCC}_{i}\right){\upbeta}_{i})}$$

With (*CCC*_*j*_*-CCC*_*i*_) > 1 and β_i_ > 2 or with (*CCC*_*j*_*-CCC*_*i*_) > 2 and *β*_*i*_ > 1, the agent i punishes the agent j with high probability. With (*CCC*_*j*_*-CCC*_*i*_) × *β*_*i*_ < 1, the agent *i* punishes the agent *j* occasionally. In this construction, a lower *CCC* agent with *β* > 2 punishes a higher *CCC* agent more accurately than a slightly lower *CCC* agent. A lower *CCC* agent with *β* < 2 punishes a higher CCC agent less accurately and may not punish a slightly lower *CCC* agent. For example, in a random pair, with (*CCC*_*j*_*-CCC*_*i*_) = 1 with *β*_*i*_ > 2 agent i punishes the agent j with a high probability close to one and *β*_*i*_ < 2 punishes rarely. Depending on the differences in *CCC* values and *β* many possibilities exist. The above considerations are different from the existing models of altruistic punishments^31,34^ and close to the experimental observations in public goods provision^12,37^. In the population of heterogeneous conditional cooperators, the lower *CCC* agents donate and punish frequently, the moderate *CCC* agents occasionally free ride and punish, and very high *CCC* agents mostly free ride and do not punish.

### Reproduction

After altruistic punishments, each agent’s total payoff equals to the sum of the payoff from the PGG and the potential costs incurred in imposing altruistic punishments *(π*_*alt*_) and the cost paid if the punishment is received *(π*_*in*_). For instance, *i*th agent’s payoff will be *π*_*i* =_ *π*_*Gi*_ + *π*_*alt*_ + *π*_*in*._ All the agents occasionally update their strategies by pairing another randomly matched agent and adapting the role model’s strategy with a probability proportional to the payoff difference^45,46^. In terms of cultural evolution each individual imitates the successful agent’s social behavior; all the agents update their CCC and *β*_*i*_ values simultaneously with certain mutations. The updating is done by the following procedure^4,44^. An agent *i* potentially imitates successful individual *j*’s social behavior (*CCC*_*j*_) and *β*_*i*_ with probability, *q*_*r*,_

4 $${q}_{r}= \frac{1}{1+\mathrm{exp}(-\left({\uppi}_{j}-{\uppi}_{i}\right){\upbeta}_{i})}$$

where (*π*_*j*_*-π*_*i*_) is the cumulative payoff difference of agents *j* and *i* respectively. *β*_*i*_ is *i*th agent’s propensity, which controls the steepness of Eq. (4). An agent with a higher *β*_*i*_ more accurately imitates the social behavior of the role model. In each generation the population undergoes 10% mutations, i.e., each individual miscopies successful role models’ properties with probability 0.1. From Eqs. (1) and (4) it suggests that the agents who are more willing to donate are also more willing to punish free riders.

### Simulations

In the simulations, the initial propensity (*β*) values are drawn from uniform distribution [1, 3] and *CCC* values are drawn from [1, *N*], where *N* is the population size = 100 similar population size is chosen elsewhere^45,47^. Agents enter into the PGG with the initial payoff (*u*) = 50 units and number of donations in the zeroth round = 10. Each agent decides their donation by using a stochastic conditional decision rule, Eq. (1). The donation cost is 1 unit and the enhancing factor of the collected donation is *α* = 3 units. The total payoff of an agent is given by Eq. (2). A generation consists of three rounds of PGG. After each generation, altruistic punishments were implemented and, subsequently, the population updates or reproduces by using Eq. (3) with 10% mutations.

Mutations are created by adding a random value, drawn from a Gaussian distribution with mean zero and *s.d*. = 5 (max = 50 and min =  − 50). Each agent in the population miscopies the role model properties with a probability 0.1. After updating by using Eq. (4), a randomly drawn *CCC* value is added to the updated *CCC* value and a *β* value is replaced by a randomly drawn value from uniform distribution of (0, 3). If the updated *CCC* value is greater than *N*, it is rounded off to *N*; if the resultant *CCC* value is negative, it is rounded off to zero. This allows the population to have unconditional free riders (*CCC* = *N*) and unconditional cooperators (*CCC* = 0).

The number of donations in the zeroth round is 10. A generation consists of three rounds of repeated PGGs without enforcing altruistic punishments. After each generation altruistic punishments were implemented and subsequently all the individuals in the population simultaneously update their strategies with 10% mutations. To reduce individual trial variations, each experimental condition (a fixed set of parameters) is run over 20,000 and iterated 20 times and the average data is used to plot the results. We observed that there is no difference in the results after the first few thousand generations; therefore, we have plotted donation fractions for the first 10,000 generations. We plotted the distribution of CCC and *β* values for different experimental conditions in the last 20,000th generation. We measured donation fractions, i.e., the fraction of donations in a specified number of generations. Asymptotic donation fractions are computed by taking donations in the last 20,000 generations of the 20,000th generation. We computed the distribution of *CCC* of the population and β values, which indicates the composition of population (or strategies). In simulations, we kept the following parameters constant: the donation cost (*u*) = 1, enhancing factor of collected donations (*α*) = 3. The probability of altruistic punishments after each generation is designated by *w*. Simulations are performed with varies *w*(*w* < 1) and inflected costs. In the model, if the punishing cost is *x*, the inflicted punishment cost will be 3*x*.

## Results

Agents starting with arbitrary *CCC* and *β* values, donating to the public good by using Eq. (1), enforcing altruistic punishments using Eq. (2), and updating strategies using Eq. (4), for certain punishment costs, meant that altruistic punishment evolved in the population. Evolution of altruistic punishments crucially depends on the inflected cost of the punishment and the opportunities given to individuals to enforce punishments.

**Figure 1:**
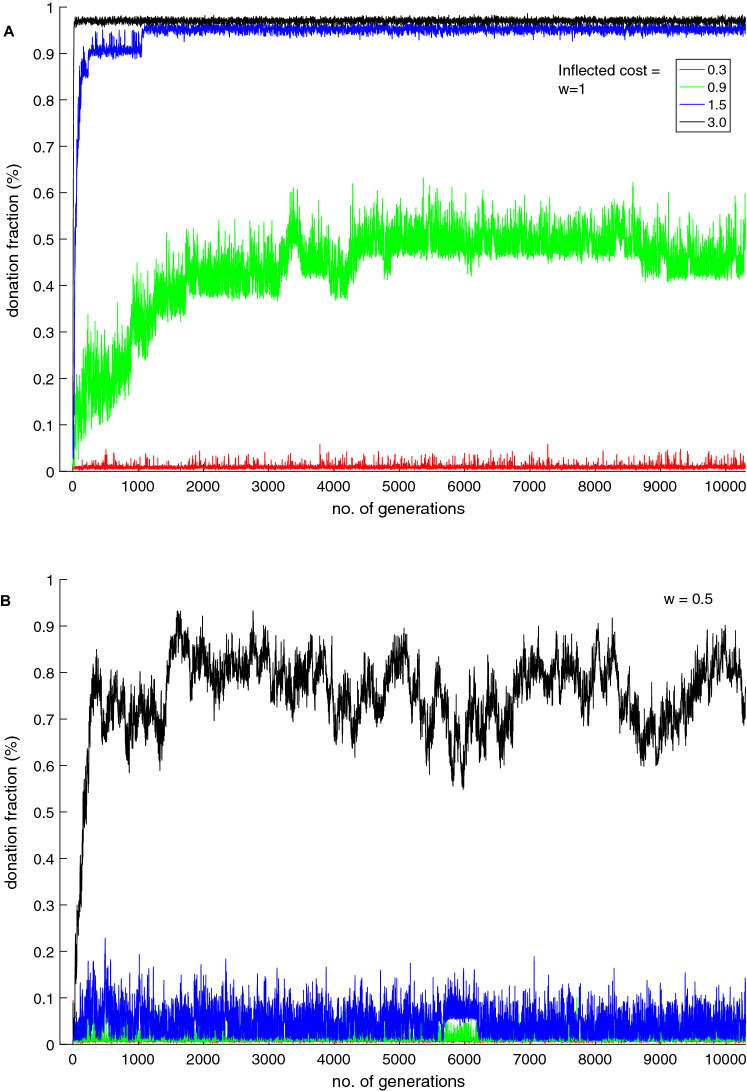
Donation rates for (**A**) (*w*) = 1 and for (**B**) (*w*) = 0.5 for various cost values for the first 10,000 generations.

Figure 1 represents donation fractions (fraction of population donated to PGG) for various inflected costs across generations with *w* = 1 and *w* = 0.5. The donations are much higher with *w* = 1 than with *w* = 0.5 for the same inflected costs. With *w* = 1, the donation fractions decline as the cost of the inflected punishment also declines.

**Figure 2:**
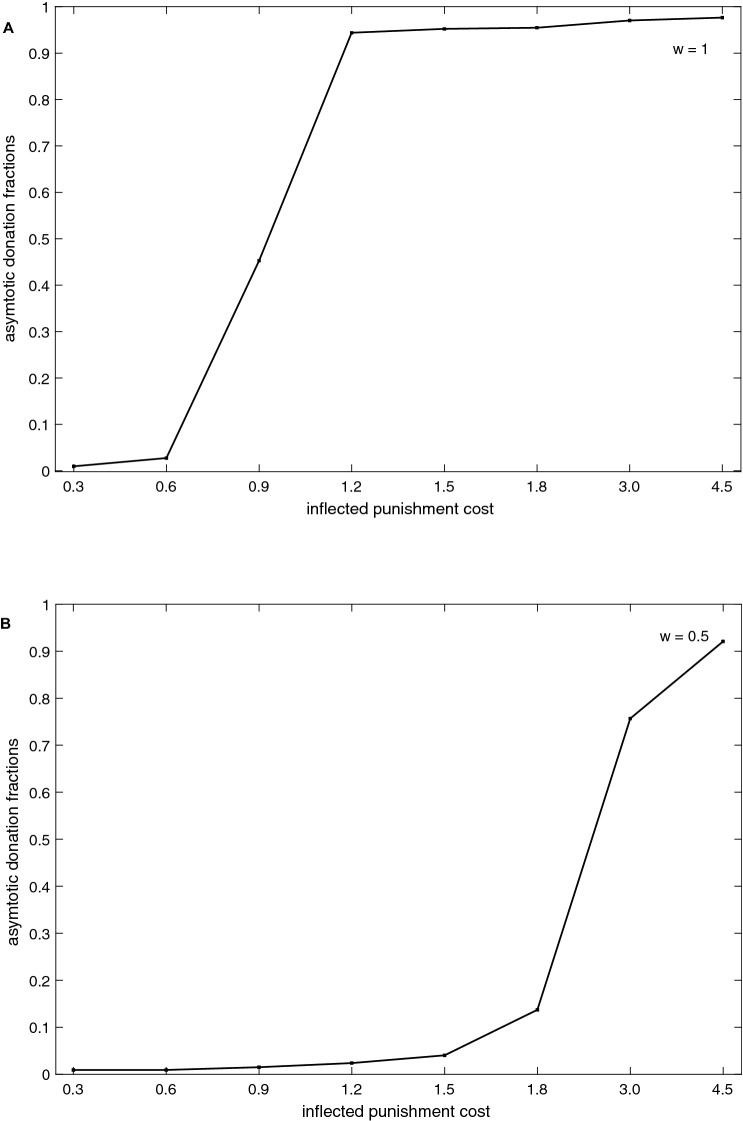
Asymptotic donations, fractions of population with various punishment costs. (**A**) (*w*) = 1 and (**B**) (*w*) = 0.5.

In Fig. 2, asymptotic donation rates are shown across different costs of inflected punishments with *w* = 1(when the agents have been given more opportunities to punish after every few rounds) and with *w* = 0.5 (when the agents punish randomly) respectively. With *w* = 1 the donation rates are higher after inflected cost > 0.9. With *w* = 0.5, the donation fractions are higher only for higher inflected costs.

**Figure 3:**
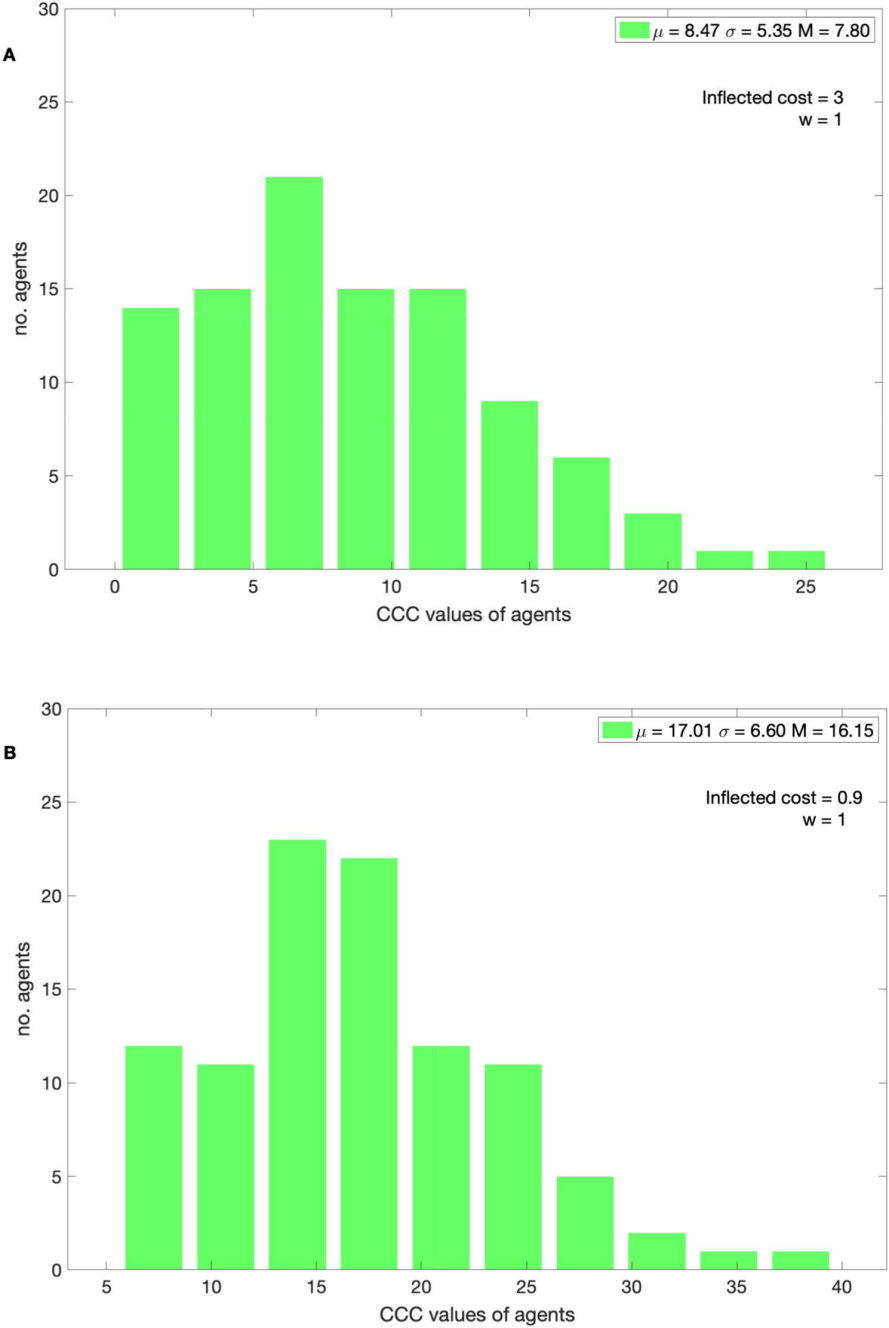
The distribution of *CCC* values in the 20,000th generation with a bin size of 10. With *w* = 1 (**A**) (cost) = 3 and with (**B**) (cost) = 0.9.

In Fig. 3, the distribution of *CCC* values of the population shown for various inflected costs. With *w* = 1 and with inflected cost = 3, the frequency of punishments is high, therefore high CCC agents are penalized substantially. The higher *CCC* agents become extinct from the population because the score of these agents is lower than the mean population score. As the generations increase, with cost = 3, the population moves towards lower *CCC* agents (mean = 8.47 and median = 7.80) and, with inflected cost = 0.9, the population moves towards moderate *CCC* value agents (mean = 17.01 and median = 16.15).

**Figure 4:**
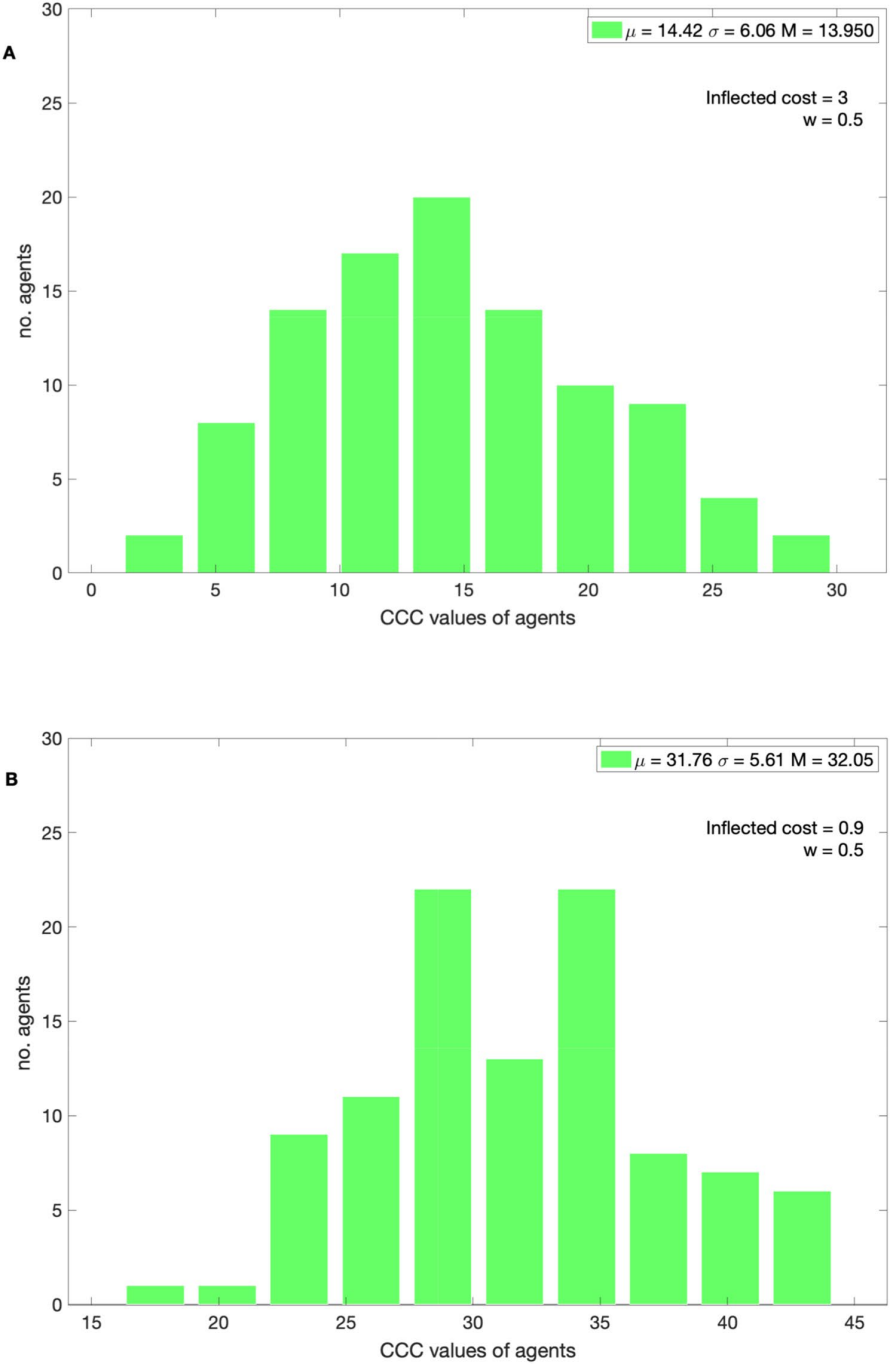
The distribution of *CCC* values in the 20,000th generation with a bin size of 10. With *w* = 0.5 (**A**) (cost) = 3 and with (**B**) (cost) = 0.9.

In Fig. 4, the distribution of *CCC* values of the population is shown for various inflected costs. The results show that lower *CCC* agents proliferate in the population with higher inflected costs, these individuals not only donate to the public good but also enforce altruistic punishments. The population remains heterogeneous.

With *w* = 0.5 and with inflected cost = 3, the frequency of punishments is low, therefore high *CCC* agents are not penalized substantially, thus, moderate *CCC* agents still remain in the population. The population moves towards moderate *CCC* agents (mean = 14.42 and median = 13.95). With inflected cost = 0.9, the population moves towards larger *CCC* value agents (mean = 31.76 and median = 32.05).

**Figure 5:**
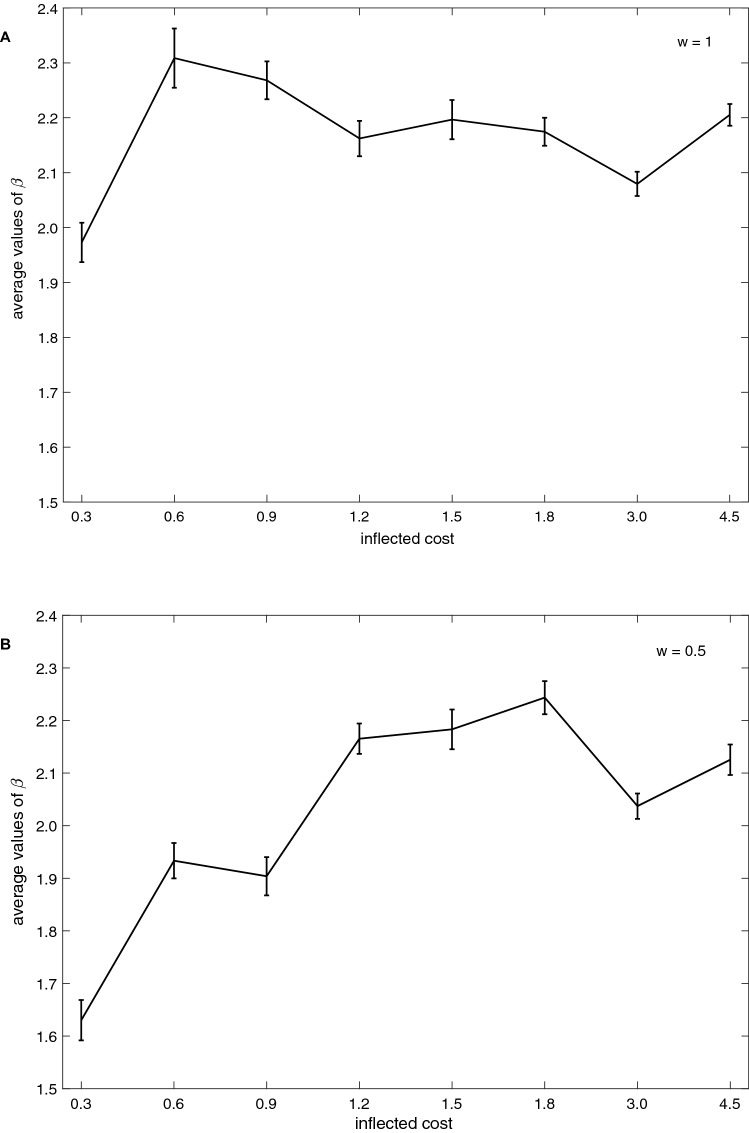
Population average *β* values for various costs in the 20,000th generation. (**A**) (*w*) = 1 and (**B**) (*w*) = 0.5.

In Fig. 5 the average *β* values of the population shown for various inflected costs with *w* = 1 and with *w* = 0.5. For higher inflected costs the average values of *β* decline, i.e., the population adapted to probabilistic donations and punishments. With *w* = 0.5, *β* values increase only for higher costs of inflected punishment; the population adapted relatively higher *β* values than in the previous condition.

## Discussion

The results of the simulations show that in a population starting with arbitrary conditional cooperative strategies, enforcing altruistic punishments based on relative differences in *CCC* values, and adapting successful role models’ social behavior, altruistic punishments evolve in the population and establish stable cooperation. Interestingly, with certain infected costs, the population is adapted to generous altruistic punishment strategies. Showing generosity towards occasional free riders allows the individuals to cooperate in the next interaction and it has been shown that generosity is a superior strategy when the well-intentioned players commit mistakes occasionally^48^. The evolution of generosity in the population crucially depends on the cost of altruistic punishment or inflicted punishments and the opportunities given to the population to enforce altruistic punishments. In other words, when the punishment cost is moderate, agents do not punish negative reciprocators (middle range *CCC* value agents) accurately but punish selfish free riders accurately (high *CCC* value agents). When punishment costs are high, agents punish selfish free riders accurately as the population is dominated by lower *CCC* agents. The recognition that conditional cooperators are adapting to generous altruistic punishments rather than strict altruistic punishments has important consequences in enforcing altruistic punishments^12,21,37,49^ and in understanding the evolution of altruistic punishments in social dilemmas^9,10^.

In the standard PGG without altruistic punishments, the free riders (high *CCC* agents) proliferate in the population because these agents score relatively higher payoffs than the reciprocators (lower *CCC* agents), thus no cooperation is established. Suppose, a population consists of pure strategies such as altruists, free riders, and reciprocators who not only cooperate but also punish the free riders, clearly the reciprocators score fewer payoffs than the altruists who donate but do not punish free riders. The situation might change when we consider the spatial structure of the population^21^. For sufficient costs of inflicted punishments, altruists proliferate in the population. In this stylistic model, the cooperation is not stable; in the sea of altruists, when an occasional mutant, a free rider, enters into the population, the free riders destroy the cooperation. Whereas, when the population consists of heterogeneous conditional cooperators, the free riders do not gain a very high payoff, thanks to altruistic punishments and the heterogeneity of the population. If an occasional free rider, a mutant, gets into the population, the cooperation is not destroyed as the population remains heterogeneous and consists of reciprocators who also act as altruistic punishers. However, the composition of the population depends on the inflected costs and number of opportunities given for the implementation of altruistic punishments. Lower inflected costs and rare opportunities to punish free riders do not establish cooperation.

In the model, with *w* = 1, all the agents have an opportunity to punish potential free riders in the population after each generation. Typically, the lower *CCC* agents potentially punish the higher *CCC* agents or potential free riders (Eq. 3). The altruistic punishments are only effective if the population punishes the free riders more frequently and occasional free riders less frequently. For certain critical inflected punishment costs, after punishments are implemented, the relative payoff of the higher *CCC* agents is less than the payoff of the lower *CCC* agents. For example, with *w* = 1 and inflected punishment cost = 3 units, the population starting with arbitrary *CCC* values, after several thousands of generations, the population is adapted to lower *CCC* values (mean = 8.47) (Fig. 3A) and also adapted to higher *β* values (Fig. 5A). With the higher *β* values and with lower *CCC* values, from the Eq. (3) the reciprocators punish the selfish free riders frequently.

When the inflected cost is lower, the population is adapted to relatively higher *CCC* and lower *β* values (see Figs. 3A and 5A), cooperation is established but is not high. In this condition if the occasional free riders or negative reciprocators were punished frequently, the population would wipe out many moderate *CCC* agents who are helpful in creating critical levels of cooperation and involved in altruistic punishments against the selfish free riders. The population does not establish conditional cooperation if the inflected costs are too low (Fig. 2A). When the punishments were enforced in a probabilistic manner, i.e., *w* < 1, the agents get rare opportunities to punish free riders, thus higher CCC agents dominate the population, and thus no cooperation is established. For the same punishment cost with *w* < 1, the population is adapted to relatively lower *β* values and higher CCC agents than the above condition (*w* = 1). With *β* > population enforces altruistic punishment,and imitates successful role models’ behavior accurately and with *β* < 2 vice-versa. For instance, with *w* = 0.5 with inflected cost = 3, the population is dominated by moderate *CCC* values (mean = 14.42, see Fig. 3B), higher *β* values (see Fig. 5B), and the cooperation levels are higher but not higher as with *w* = 1. In this condition population is adapted to generous punishment strategies. With *w* = 0.5 and with lower inflected cost = 0.9 (see Fig. 2B), the donation levels are close to zero and the *CCC* values of the population are higher (mean = 31.76) with cost = 0.9. Switching punishments on and off (*w* = 0.5) does not reduce the payoff of the higher CCC agents (free riders) substantially, therefore moderate or higher *CCC* agents dominate the population, thus no cooperation is established and such behavior has been observed in experiments elsewhere^50^. In summary, with higher inflected cost and *w* = 1 the population able to establish conditional cooperation and the population adapted to higher *β* and lower CCC value agents. Thus, the population is adapted to strict altruistic punishments. Interestingly, with *w* = 0.5 and with higher inflected cost or with *w* = 1 and with moderate inflected costs, the population is adapted to relatively lower *β* and moderate *CCC* values; the population is adapted to generous altruistic punishments.

In the model, the occasional mutations do not destroy the conditional cooperation when high inflected cost, high *w,* and with a moderate *w*. In these conditions, for instance, a mutant *CCC* = *N*(= 100) gets into population, with *w* = 1 and sufficient inflected cost, the mutant will be punished quickly as the population is dominated by lower *CCC* agents. On the other hand, if the population consists of only altruists (*CCC* = 0) and suppose an occasional mutant happens to be an unconditional free rider (*CCC* = 100)), the mutant can take over the population because free riding gives more advantages than the population average.

The proposed model is placed in the generic settings of conditional cooperation with considering spatial structure, yet some aspects of the model do not eliminate its few shortcomings. For example, an agent who reciprocated in PGG can be punished by other reciprocators in the group because the altruistic punishments are implemented by random pairings and based on relative *CCC* values, not based on the actions of individuals. As noted in the literature, free riders can bribe the altruistic punishers^51^ and free riders can be involved in punishing^20,21^. Punishing reciprocators or negative reciprocators might lead to retaliation against the reciprocators or free riding in the next round. However, in the current model such occasions are rare in the long run because the difference in *CC*C values of reciprocators is small and *β* is not very high. The model does not consider other psychological aspects of agents such as ‘warm glow^52^’ the influence of partners and strangers^10^, and beliefs about other agents^38^. Further, it is worthwhile to consider conditional punishments^28^, class dependent punishment strategies^24^, and sharing responsibility of punishing^25^ in further research.

In summary, the consideration that individuals in repeated public good games behave like conditional co-operators—i.e. individuals who are more willing to donate are also more willing to punish free riders—, and that these conditional cooperators are heterogeneous, throws new light on the evolution of altruistic punishments in a heterogeneous population. The essential feature of conditional cooperation rests on the population’s ability to create a critical amount of cooperation in the initial rounds and to create conditions in which reciprocators cooperate and establish cooperation. The simulations show that when conditional cooperators make occasional mistakes in their punishment strategies, evolution prefers generous altruistic punishment strategies rather than strict punishment strategies; these strategies not only save the overall payoff of the group but also help to initiate cooperation from conditional cooperators. Unlike previous models^31–34,53^ and not considering spatial structure^20,21,24,25,28^, the current model provides new mechanisms to establish conditional cooperation and the evolution of altruistic punishments. Conditional cooperation operates with reciprocity, fairness, and retaliation^7^ but without using payoff maximizing strategies^13^. The model shows that to establish cooperation in public good scenarios, implementing stern altruistic punishments against negative reciprocators is not required but it is necessary to punish selfish free riders to create sufficient cooperation in each round and build cooperation in the subsequent rounds. By punishing free-riding in the initial rounds one can facilitate conditions to establish cooperation but also help to stabilize cooperation by using feedback loops^54,55^ (Supplementary Information).

## Supplementary Information

Supplementary Information 1.

Supplementary Information 2.

## Data Availability

The MATLAB code that supports this study's findings is available in the Supplementary Information.
