# Supplementary material for: Evolution of altruistic punishments among heterogeneous conditional cooperators
Source: Sci Rep. 2021 May 18;11:10502. doi: 10.1038/s41598-021-89563-z (PMC8131352; doi:10.1038/s41598-021-89563-z)
Supplement: Supplementary file 1 — Supplementary Information 1. [file 41598_2021_89563_MOESM1_ESM.pdf]

All the following program files kept in the same folder

The function “ PGG_Alt_Punish.m” is a Matlab file implementation of the model.

The program file, “PGG_Alt_Punish_RUN.m” is for creating data using PGG_Alt_Punish.m function.

The program file, “randnlimt.m” is for creating mutations and used by PGG_Alt_Punish.m.

The data and plotting program, “PGG_Punish_plotting_figures.m” is available in the folder PGG_Punish_Supp_R
